# Unravelling the effects of radiation forces in water

**DOI:** 10.1038/ncomms5363

**Published:** 2014-07-07

**Authors:** Nelson G. C. Astrath, Luis C. Malacarne, Mauro L. Baesso, Gustavo V. B. Lukasievicz, Stephen E. Bialkowski

**Affiliations:** 1 Departamento de Física, Universidade Estadual de Maringá, Maringá, Paraná 87020-900, Brazil; 2 Department of Chemistry and Biochemistry, Utah State University, Logan, Utah 84322-0300, USA

## Abstract

The effect of radiation forces at the interface between dielectric materials has been a long-standing debate for over a century. Yet there has been so far only limited experimental verification in complete accordance with the theory. Here we measure the surface deformation at the air–water interface induced by continuous and pulsed laser excitation and match this to rigorous theory of radiation forces. We demonstrate that the experimental results are quantitatively described by the numerical calculations of radiation forces. The Helmholtz force is used for the surface radiation pressure. The resulting surface pressure obtained is consistent with the momentum conservation using the Minkowski momentum density expression assuming that the averaged momentum per photon is given by the Minkowski momentum. Considering the total momentum as a sum of that propagating with the electromagnetic wave and that deposited locally in the material, the Abraham momentum interpretation also appears to be appropriate.

## Abstract (web summary)

Contradictory theories are used to describe radiation pressure at the interface between air and dielectric liquids. Here, Astrath *et al*. measure the surface deformation of water by laser excitation and conclude that both theories with different forms of electromagnetic momentum tensor may be correct.

## Graphical abstract

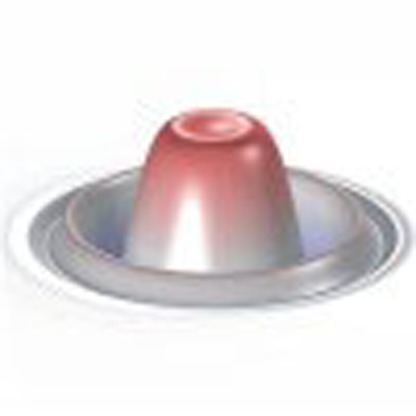

The effects of radiation pressure exerted on a dielectric surface exposed to electromagnetic radiation can be interpreted as the transfer of momentum from photons at the surface parallel to the propagation of the incident electromagnetic radiation. Radiation pressure effects were predicted by Maxwell in 1871 (ref. 1) and experimentally observed by Lebedew in 1900 (ref. 2). In 1905, Poynting^3^ presented a detailed geometrical calculation of the force by radiation pressure of light incident from free space on a transparent and non-dispersive dielectric medium, which predicted an outward force normal to the surface of the dielectric opposite to the direction of propagation of the incident electromagnetic field. Conflicting theories for the energy–momentum tensor were proposed by Minkowski in 1908 (ref. 4) and Abraham in 1909 (ref. 5) to explain this effect. These have subsequently been extensively debated in the literature over the past century^6^,^7^,^8^,^9^,^10^,^11^,^12^,^13^,^14^. The Minkowski representation predicts a momentum producing an outward surface force in the medium proportional to its refractive index, *n*, as *p*_M_=*np*_0_, in which *p*_0_=*U*/*c* is the photon momentum in the vacuum, *U* is the energy of light and *c* is the speed of light. In contrast, the Abraham representation leads to a momentum within the medium in the form *p*_A_=*p*_0_/*n*, which in turn produces an inward force to the medium.

At the theoretical level, the Minkowski–Abraham controversy has apparently been resolved by identifying the Abraham momentum as the kinetic momentum and the Minkowski momentum as the canonical momentum^6^. Under this reconciliation, there can be no discrepancy between Abraham and Minkowski formulations, provided sufficient care is taken in the treatment of all relevant forces^13^,^15^,^16^. Yet there has been so far only limited experimental tests^6^,^17^ of our understanding of radiative transfer between electromagnetic radiation and dielectric media. This is, in fact, of great importance when the problem defied conclusive theoretical description for almost a full century. However, these forces have been described in details^18^,^19^,^20^,^21^ and can qualitatively describe some of the most discussed experimental data in the literature^22^,^23^.

In the classical experiment of radiation pressure of Ashkin and Dziedzic^23^, for instance, normally incident tightly focused laser pulses generated deformations of air–water interface, and it was found that liquid surface experienced a net outward force (Minkowski momentum) regardless of the direction of laser propagation. As described by Gordon^13^ and Loudon^19^, the expansion of the liquid was caused by radial forces (electrostriction force) acting towards higher field strength causing a load pressure increase in the centre of the laser beam (toothpaste-tube effect). The experiment presented in the current research is a significant advance over this important contribution of Ashkin and Dziedzic^23^. It tests our understanding of dynamics and momentum transfer in coupled electromagnetic/dielectric systems, which is greatly needed, given the historical difficulty involved in understanding these systems.

We model the effects of these radiation forces using finite element analysis (FEA) and show that the experimental results are in quantitative agreement with both Minkowski and Abraham theoretical representations. Radiation forces are quantitatively described by the Helmholtz electromagnetic force density. We use the photomechanical mirror (PM) method to measure the time-dependent nanometre-scale deformation generated on the water surface due to the radiation forces by exciting the sample with continuous or pulsed lasers.

## Results

### Photomechanical mirror

**Figure 1:**
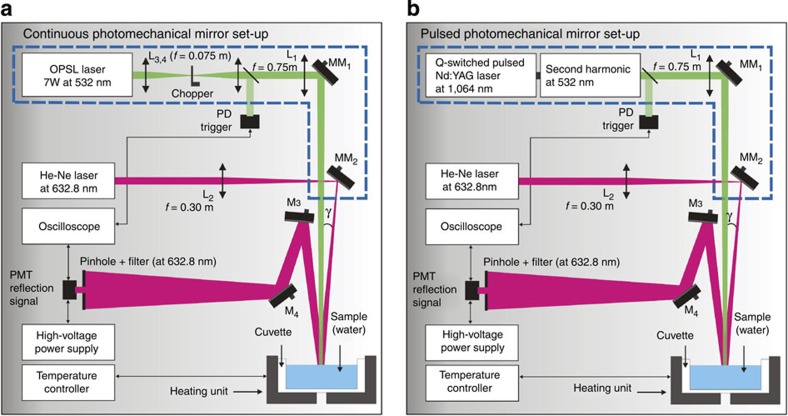
PM set-up. Schematic diagram of the apparatuses for time-resolved PM under continuous (**a**) and pulsed (**b**) experiments. M_i_, MM_i_ and L_i_ stand for mirrors, motorized mirrors and lenses, respectively. PD and PMT stand for photodiode and photomultiplier tube, respectively. The apparatuses depicted within the delimitated dashed lines were mounted in separated actively damped optical tables to prevent mechanical vibration on the water surface. A complete experimental description is presented in the Methods section.

The PM method has been used to detect surface displacement of a few nanometres in solids^24^,^25^,^26^,^27^,^28^. One laser irradiates the sample normal to its surface and a low-irradiance laser probes the deformation of the sample by measuring the on-axis intensity variation of the central portion of the probe beam reflected off of the sample surface; the expansion/contraction of the sample diverges/converges the probe beam at the detector, diminishing/increasing the signal at the detector. The experimental apparatuses used in this work are described in Fig. 1a,b for continuous and pulsed excitation, respectively.

**Figure 2:**
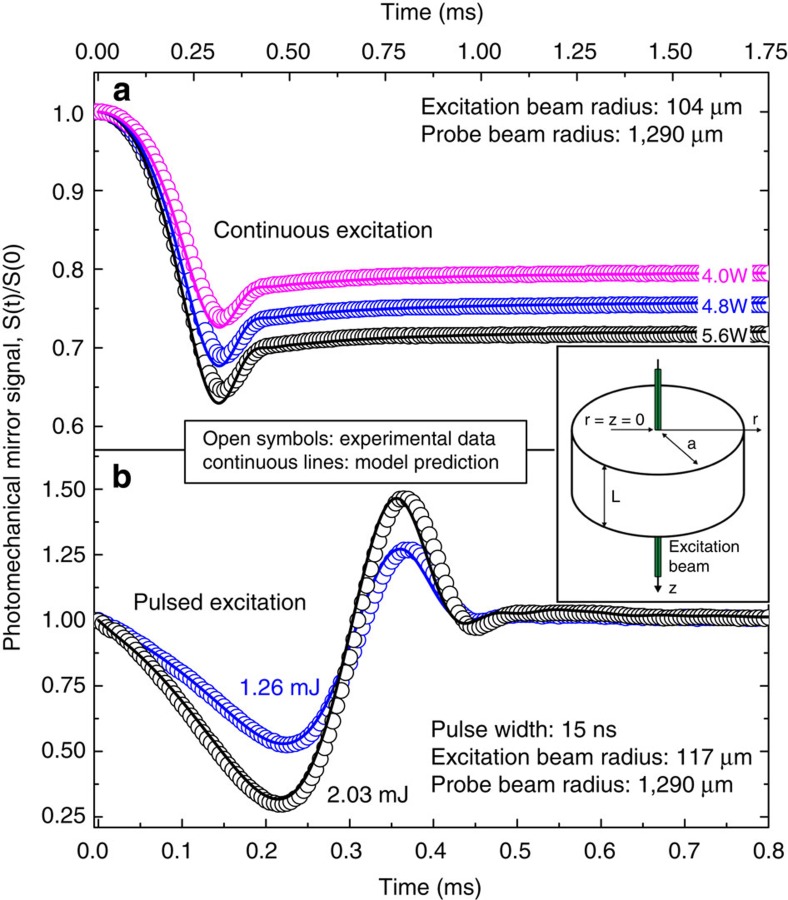
Time-resolved PM transients. PM signal under (**a**) continuous and (**b**) pulsed laser excitation at 532 nm. The transients show the intensity variation of the centre of a continuous probe beam laser reflected off of the water surface measured at the PMT positioned in the far field. Open symbols are experimental data and continuous lines represent the numerical calculations using *S*(*t*)/*S*(0), in which *S*(0) is the signal at *t*=0. The error bars for the experimental data are smaller than 0.2%.

Milli-Q water was used in the experiments. The sample was placed in a cylindrical quartz cuvette of radius *a*=30 mm and *L*=8 mm high, as illustrated in the inset of Fig. 2. The sample temperature was (298.15±0.01) K. More than 100 transients were averaged and results for the PM signals under continuous and pulsed laser excitations at 532 nm are presented in Fig. 2. The transients show the intensity variation of the centre of a continuous probe beam laser reflected off of the water surface measured at the photomultiplier tube (PMT) positioned in the far-field. Power, energy and laser beam dimensions are listed in the figures.

Figure 2a shows three PM transient signals under continuous excitation for different excitation powers. The probe beam intensity decreases with time due to elevation, that is, a convex mirror-like optical element of the water surface for a duration less than 200 μs. Subsequently, a reduction in the signal towards a steady state is observed. This reduction is discussed below. For the pulsed excitation (Fig. 2b), two transients are presented for different energy levels. The radiation force exerted in the water by the pulse is much shorter than the transient signal (pulse width was 15 ns). The PM sensor is measuring, in fact, the surface wave propagating after the laser pulse.

The deformation of the sample surface, *u*_z_(*r*, *z*=0, *t*), produces a phase shift to the reflected part of probe beam given by Φ(*r*, *t*)=(4*π*/*λ*_p_) *u*_z_ (*r*, *z*=0, *t*)^26^, where *λ*_p_ is the probe beam wavelength. Considering only the centre of the probe beam spot at the detector plane in the far-field region, and using Fresnel diffraction theory, the relative far-field intensity signal *S*(*t*) results in Sato *et al*.^26^


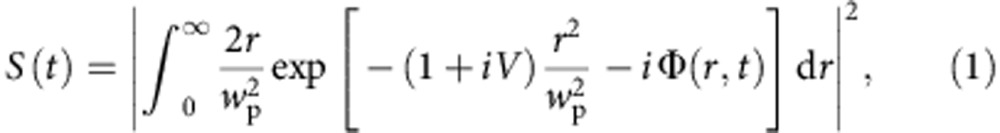


where *V*=*Z*_1_/*Z*_C_+*Z*_C_[(*Z*_1_/*Z*_C_)^2^+1]/*Z*_2_, *Z*_C_ is the confocal distance of the probe beam, *Z*_1_ is the distance from the probe beam waist to the sample, *Z*_2_ is the distance between the sample and the detector and *w*_p_ the radius of the probe beam at the sample surface. The experimental parameters are listed in Supplementary Table 1. Equation (1) can be evaluated numerically. The calculation of *S*(*t*) requires the determination of *u*_z_ (*r*, *z*=0, *t*) considering all the effects of the radiation forces in the liquid.

### Forces at a dielectric interface

Landau and Lifshitz^29^ give the body force in terms of the stress tensor *σ*_ik_ and the momentum density *G*_i_ in the form


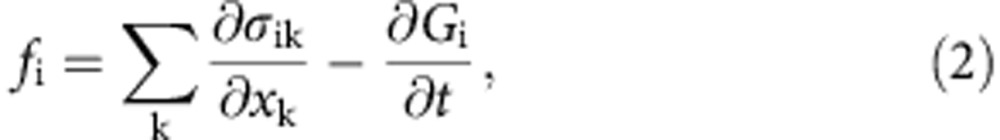


with *σ*_ik_ for a fluid in the absence of free charge and current given by


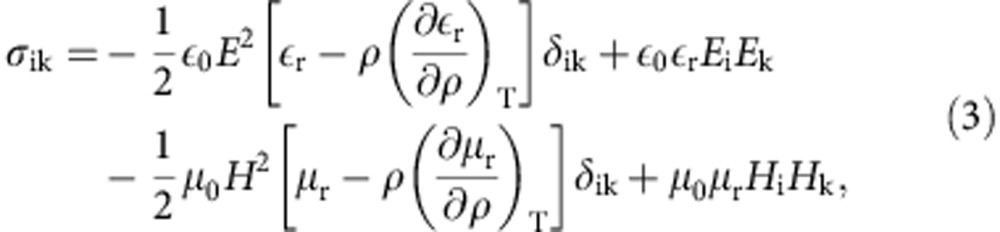


and the momentum density **G**=**E** × **H**/*c*^2^. The first term in the stress tensor accounts for electrostriction and the third term for magnetostriction. The required force, making use of above relations and the Maxwell’s equations, and assuming a dielectric fluid, is therefore^5^,^13^,^29^


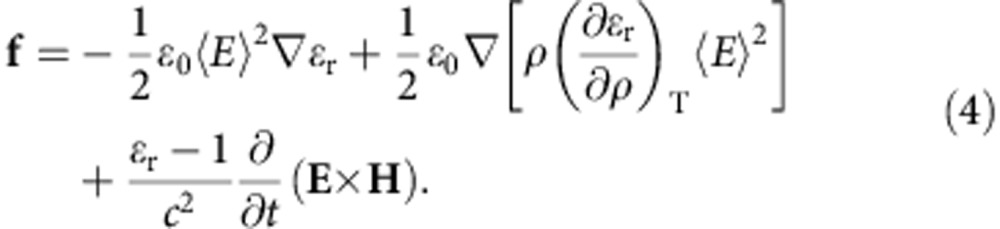


*ρ* is the mass density, *c* is the speed of light, **E** and **H** describe the electric and magnetic fields, respectively, *ε*_0_ and *μ*_0_ are the permittivity and permeability in vacuum, respectively, and *ε*_r_=*ε*/*ε*_0_ and *μ*_r_=*μ*/*μ*_0_ are the relative permittivity and permeability of the medium, respectively. The first term in equation (4) is a common term arising from the Minkowski and Abraham energy–momentum tensors and is often called the Minkowski–Abraham force acting where relative permittivity presents spatial variation, especially in interfaces where *ε* presents discontinuity. The second term is the electrostriction force and it is important when the field and dielectric permittivity are inhomogeneous. The last term is known as the Abraham force density. The existence of this term was demonstrated in experiment with quasistationary field^30^. The latter term averages to zero at optical frequencies and can be neglected in our model. In the absence of Abraham force term, equation (4) reduces to the Helmholtz force^29^,^31^.

For a laser beam normally incident from free space (air) on a flat surface of a dielectric liquid, after a few nanoseconds the volume contribution of the electrostriction is cancelled out by its surface contribution^32^. The surface motion timescale is much longer than this initial transient and surface deformation is described by that due to the Minkowski–Abraham term as well as those due to gravity and surface tension^32^,^33^. This result could be obtained from the Helmholtz force, as showed below.

The total surface force, *f*_s_, for a laser beam normally incident from air on the water, takes only the azimuthal component of the gradient, ∇_z_=∂/∂*z*, as


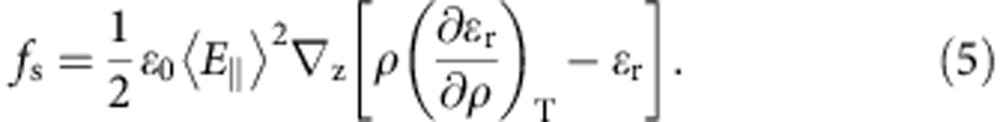


Here ‹*E*_||_›^2^=*T*‹*E*_inc_›^2^ is the electric field tangential to the surface of the water and can be written in terms of the transmission coefficient, *T*=4*n*/(*n*+1)^2^, and the incident electric field, ‹*E*_inc_›^2^. The pressure imparted by this surface force can be obtained by integrating the normal component of the force across the interface air–water, that is, 
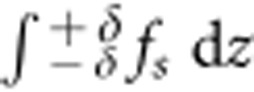
 with *δ*→0. Integration results in a pressure *P*_in_ pushing the surface inwards, which is compatible with the Abraham momentum,


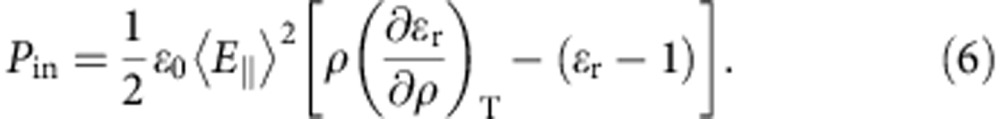


This pressure is counterbalanced by the hydrostatic pressure *P*_out_ due to electrostriction^13^,^34^. This volume force *f*_V_ is written as a radial gradient, ∇_*r*_=∂/∂*r*, of *P*_out_ as *f*_V_=∇_r_*P*_out_ . From equation (4), *f*_V_ is


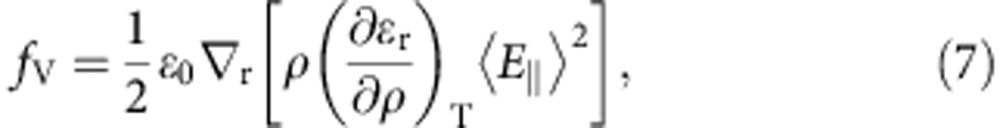


and *P*_out_ is thus given by


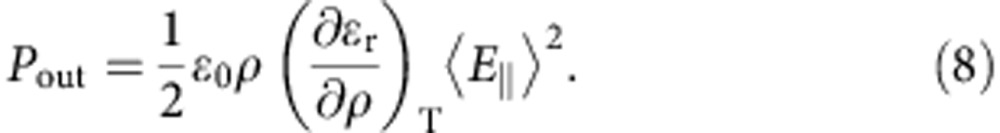


Using the well-known relation for the field intensity, *I*(*r*, *t*)=*ε*_0_*cn*‹*E*_inc_›^2^ , the overall pressure that elevates the surface of the liquid is^34^


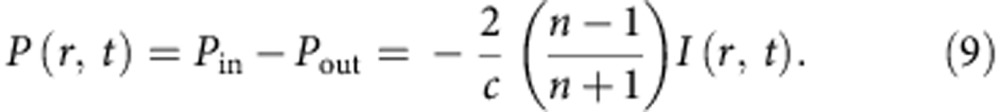


The sign of this pressure is an outward pressure effectively expanding the fluid. This is equivalent to assuming that the averaged momentum per photon is given by the Minkowski momentum or canonical momentum^13^ as the total propagating momentum. Equation (9) is equivalent to assuming that the averaged momentum per photon is given by the Minkowski momentum or canonical momentum^13^, **K**=*ε*_r_**G**=**G**+**M**, as the total propagating momentum. Here **G**=(1/*c*^2^)**E** × **H** is the electromagnetic momentum density (Abraham momentum) and **M**=(*ε*_r_−1)**G** is the accompanying mechanical momentum of the medium. The momentum conservation at the interface air–dielectric is (1−*R*) **G**_0_=**G**+**M**+Δ**p**=*ε*_r_*T***G**_0_+Δ**p**, in which Δ**p** is the net change of momentum for a surface element during the irradiation time, that is, the pressure imparted to the medium by the field, *P*(*r*, *t*) , as in equation (9). The reflection and transmission coefficients are *R*=(*n*−1)^2^/(*n*+1)^2^ and *T*=4*n*/(*n*+1)^2^ , respectively, and **G**_0_ is the electromagnetic momentum density in vacuum. Taking all radiation pressure effects into account, the surface pressure at the dielectric boundary is consistent with the Minkowski momentum.

The pressure *P*(*r*, *t*) acts on the surface at *z*=0 parallel to the excitation beam. For our Gaussian beams, the intensity distributions are *I*_cw_(*r*)=[2*P*_e_/(*πw*_e_^2^)]exp(−2*r*^2^/*w*_e_^2^) and *I*_pulsed_(*r*, *t*)=[2*Q*/(*t*_0_*πw*_e_^2^)]exp(−2*r*^2^/*w*_e_^2^)exp[−(*t*−*ξ*)^2^/*τ*^2^]^28^. *τ* is the pulse width, *ξ* is the time to the maximum irradiance for the Gaussian pulse, 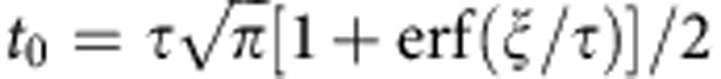 is a normalization parameter, *Q* and *P*_e_ are the pulse laser energy and continuous laser power, respectively, and *w*_e_ is the radius of the excitation beam in the sample.

### Surface deformation due to radiation forces

The effects of the radiation force on the surface displacement, in the absence of thermal effects caused by the laser absorption in the liquid, can be calculated by solving the Navier–Stokes equation with appropriated boundary conditions. We applied the FEA method for the numerical calculations using the software Comsol Multiphysics 4.3b. The ‘Laminar Two-Phase Flow, Moving Mesh’ module was used to solve the Navier–Stokes equation for incompressible flow. A complete FEA description is presented in the Methods section.


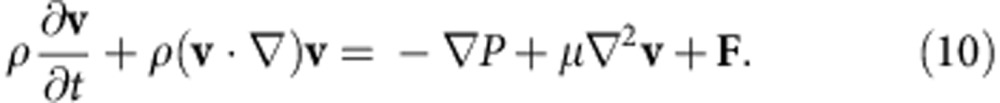


**v** describes the flow velocity, *P* is the pressure, *ρ* is the fluid density, *μ* is the dynamic viscosity and **F** is the volume force. This method is used to model two fluids separated by a fluid interface and where the moving interface is tracked in detail, including surface curvature and surface tension forces. The moving mesh method solves the flow equations on a moving mesh with boundary conditions to represent the fluid interface. In this case, additional equations are solved for the mesh deformation by means of the arbitrary Lagrangian–Eulerian method. The model was built in the two-dimensional (2D) axisymmetric geometry. The external pressure and surface tension acts on the boundary condition of the free surface. The gravity vector enters the force term as 
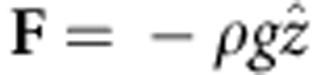
 with *g*=9.79 ms^−2^ (as measured locally). Realistic sample geometry was considered (inset of Fig. 2, *a*=30 mm and *L*=8 mm). The surface displacement along the *z* direction, *u*_*z*_(*r*, *z*=0, *t*), is calculated and the results used to generate the numerical simulations for the PM signal (equation (1)). The physical parameters of water used in the simulations are shown in Supplementary Table 2.

Figure 2 shows the calculated PM signals (continuous lines). Note that the numerical predictions are in excellent agreement for both the continuous and pulsed excitation transients. In fact, it shows quantitatively that the effects of radiation forces in water can be fully described by equation (9).

**Figure 3:**
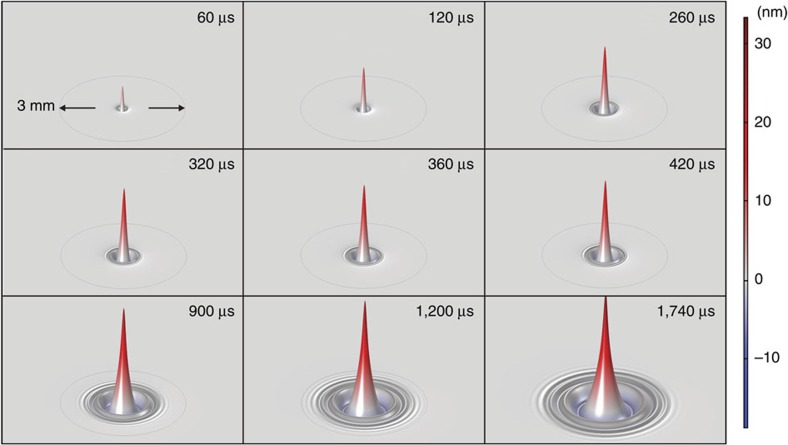
Time evolution of water surface deformation under continuous excitation. The excitation beam radius and power were *w*_e_=104 μm and *P*=5.6 W, respectively. A time evolution animation of the surface deformation can be seen in Supplementary Movie 1.

**Figure 4:**
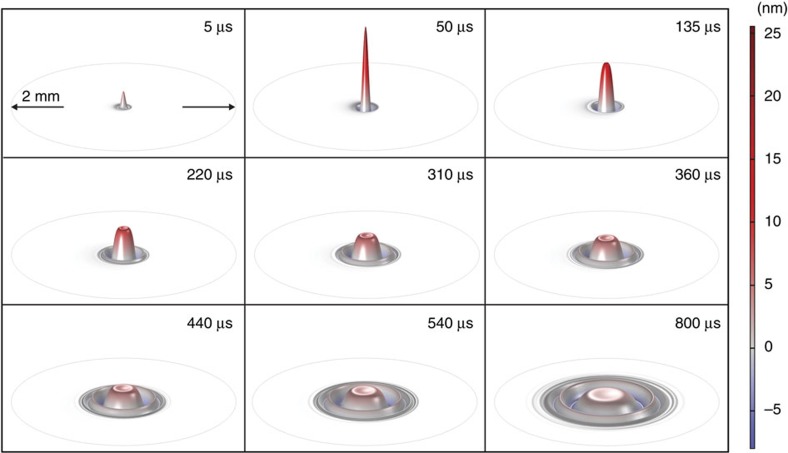
Time evolution of water surface deformation under pulsed excitation. The excitation beam radius and energy were *w*_e_=117 μm and *E*=1.26 mJ, respectively. A time evolution animation of the surface deformation can be seen in Supplementary Movie 2.

The complex form of the waves created in the water during laser excitation can be calculated using three-dimensional (3D) simulation in Comsol. Figures 3 and 4 display the actual deformation of water at different exposure times.

## Discussion

Under continuous excitation (Fig. 2a), the liquid surface rises with time reaching a maximum deformation of around 30 nm at the centre of the excitation beam. The propagation of the symmetric waves also contributes to the convoluted intensity signal observed at the detector.

For the pulsed excitation, a sharp peak appears a few microseconds after irradiation and is subsequently dispersed on the surface. Indeed, the probe beam senses the entire region affected by the excitation laser. The complex reflection pattern of the probe beam just out of the sample propagates to the detector plane. The intensity variation measured at the centre of the probe beam in the far-field has a convoluted contribution from all the surface waves created on the water, leading to the PM signal observed in Fig. 2b.

The probe laser is reflected off the water surface and spherical surface deformation causes focusing or defocusing of the central portion of the probe. The convex deformation is similar to a convex mirror in turn causing the intensity of the probe laser to decrease in far field while a concave deformation focuses the probe and thereby increases the power that passes through the pinhole placed in front of the detector. This reasoning is consistent with the signals shown in Fig. 2. In the continuous irradiation experiment, the calculated surface distortion shown in Fig. 3 is always convex and the corresponding signal shows a decrease in probe power past the pinhole at all times. As illustrated in Fig. 4, during pulsed irradiation, the surface first produces a convex column. The column subsequently collapses after irradiation causing a concave surface perturbation. This behaviour corresponds to the probe laser power initially decreasing then increasing past the pinhole. This is as observed in the experimental data.

It is clear that the numerical calculations are in excellent agreement with our experimental results, in a test that is significantly more discerning than the earlier related experiment of Ashkin and Dziedzic. This demonstrates that the system is extremely well modelled by our present understanding of radiative force transfer. The expression used for the imparted pressure on the surface of the liquid from the Helmholtz force density (equation (4)) has the same form as that using Minkowski momentum. A correct field momentum density needs to be considered for the momentum of the electromagnetic wave. The total momentum is a sum of the momentum that propagates with the electromagnetic wave, the Abraham momentum, and that which is deposited locally in the material.

## Methods

### Experiment

The time-resolved PM used in this work is illustrated in Fig. 1. Either a continuous, Fig. 1a, or a pulsed, Fig. 1b, excitation are employed in the experiments. For the continuous excitation, a TEM_00_ optically pumped semiconductor laser (Coherent, Verdi G7, 532 nm) was used to pump the sample. For the pulsed configuration, a Q-switched pulsed Nd:YAG with second harmonic TEM_00_ laser operating at 532 nm (Quantel, Brilliant B) with a pulse width of 15 ns was used to pump the sample. In both set-ups, the excitation beams were focused on the sample surface using a *f*=0.75 m focal length lens (L_1_). A 30-mW continuous TEM_00_ He-Ne laser at 632.8 nm (Melles Griot, Model 25-LHR-151-249), almost collinear to the excitation beam (*γ*<1°), focused by lens L_2_ (*f*=0.30 m), was used to probe the deformation of the sample surface. The intensity variation of the probe beam centre after reflection was detected by a pinhole-laser line filter-photomultiplier (PMT) assembly in a far field (~\n5 m from the sample surface). The laser line filter is used to prevent the excitation laser beam and ambient light from being detected by the PMT (Hamamatsu, Model R928). The PMT was biased with a high-voltage power supply (Newport, Model 70706). A digital oscilloscope (Tektronix, Model DPO4102B) recorded the data. Partial reflections from the excitation beams were used to trigger the oscilloscope by the photodiode PD (Newport, Model 818-BB-22) at a repetition frequency of 10 Hz for the pulsed experiments and 100 Hz for the continuous. A mechanical chopper (Thorlabs, Model MC2000) was used to modulate the continuous excitation. To eliminate mechanical vibration on the water surface, the excitation lasers, chopper and the motorized (Thorlabs, Model ZST213) alignment mirrors (MM_1_ and MM_2_) were placed in separated actively damped optical tables, as shown in the details (dashed lines). A heating unit and a temperature controller (Lakeshore, Model 340) were used to set the sample temperature to (298.15±0.01) K . The excitation and probe beam radii were measured with a beam profiler (Thorlabs, Model BP104-UV) and a beam profile camera (Coherent, Model Lasercam HR). Laser energy and power were measured using a pyroelectric energy sensor (Thorlabs, Model ES120C) and a power meter (Spectra-Physics, Model 407A), respectively.

### Finite element analysis

The FEA software provides numerical solutions to the Navier–Stokes equation with realistic boundary conditions imposed by the experimental geometry. The Comsol Multiphysics 4.3b software in ‘Laminar Two-Phase Flow, Moving Mesh’ module solves the Navier–Stokes equation for incompressible flow given as


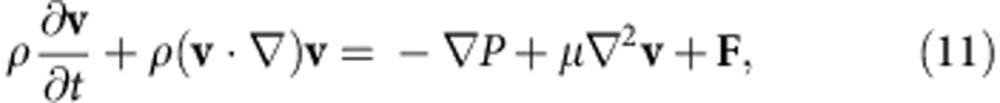


in which **v** is the flow velocity, *P* is the pressure, *ρ* is the fluid density, *μ* is the dynamic viscosity and **F** is the volume force. This method is used to model two fluids separated by a fluid interface and where the moving interface is tracked in detail, including surface curvature and surface tension forces. The moving mesh method solves the flow equations on a moving mesh with boundary conditions to represent the fluid interface. In this case, additional equations are solved for the mesh deformation by means of the arbitrary Lagrangian–Eulerian method.

FEA modelling consists of drawing the sample geometry and specifying material, boundary conditions and volume forces. The problem is first solved with rough finite element definition and subsequent refinement of elements and domain are made. The element mesh is refined until model results become independent of mesh size. Finally, **v**(*r*, *z*, *t*) can be obtained either at a single time or over a time series.

The model was built in the 2D axisymmetric geometry. There are three types of boundaries in the model domain, one boundary representing the axis of symmetry, two boundaries are modelled with no slip conditions and one free surface on which the external pressure and surface tension act. The gravity vector enters the force term as 
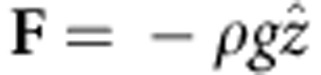
 with *g*=9.79 ms^−2^ (as measured locally). Realistic sample geometry was considered (inset of Fig. 2, *a*=30 mm and *L*=8 mm). The surface displacement along the *z* direction, *u*_z_(*r*, *z*=0, *t*), is calculated and the results are used to generate the numerical simulations for the PM signal (equation (1)). The imparted pressures under continuous and pulsed excitations are described by


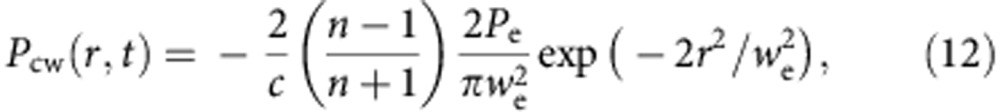


and


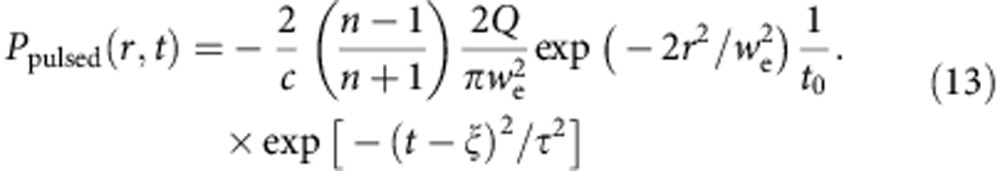


*τ* is the pulse width, *ξ* is the time to the maximum irradiance for the Gaussian pulse, 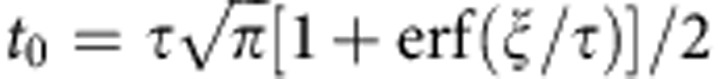 is a normalization parameter, *Q* and *P*_e_ are the pulse laser energy and cw power, respectively, and *w*_e_ is the radius of the excitation beam in the sample (constant along the sample thickness).

## Author contributions

N.G.C.A., L.C.M. and G.V.B.L. conducted the experiments; N.G.C.A, L.C.M., G.V.B.L. and M.L.B. analysed the experimental data; N.G.C.A, L.C.M., G.V.B.L., M.L.B. and S.E.B. conceived the experiments; N.G.C.A wrote the main manuscript text; S.E.B. critically reviewed and corrected the manuscript; all co-authors participated in discussions over the results and commented on the original manuscript.

## Additional information

**How to cite this article:** Astrath, N. G. C. *et al*. Unravelling the effects of radiation forces in water. *Nat. Commun.* 5:4363 doi: 10.1038/ncomms5363 (2014).

## Supplementary Material

Supplementary Information Supplementary Tables 1-2

Supplementary Movie 1 Time evolution of water surface deformation under continuous excitation. The excitation beam radius and power *w*_*e*_=104 μ*m* were and *P*_*e*_=5.6 *W*, respectively.

Supplementary Movie 2 Time evolution of water surface deformation under pulsed excitation. The excitation beam radius and energy were *w*_*e*_=117 μ*m* and *Q*=1.26 *mJ*, respectively.
