# Supplementary material for: Unravelling the effects of radiation forces in water
Source: Nat Commun. 2014 Jul 7;5:4363. doi: 10.1038/ncomms5363 (PMC4102109; doi:10.1038/ncomms5363)
Supplement: Supplementary Information — Supplementary Tables 1-2 [file ncomms5363-s1.pdf]

Supplementary Table 1

TABLE 1. Photomechanical Mirror experimental parameters.

Parameters	Unit	Continuous excitation	Pulsed excitation
Z_1	*mm*	284	284
Z_2	*m*	4.87	4.87
Z_C	*mm*	9.9	9.9
V		30.4	30.4
ξ	*ns*		30
τ	*ns*		15
w_p	μm	1290	1290
w_e	μm	104	117

Supplementary Table 2

TABLE 2. Physical properties of water used in the simulations.

Parameters		Unit	Value
ρ	Mass density	$kg\,m^{-3}$	998.2
μ	Dynamic viscosity	$10^{-3}\,s\,Pa$	0.893
σ	Surface tension	$10^{-3}\,N\,m^{-1}$	72
n	Refractive index		1.33
